# Ferrocene-1-carbaldehyde 4-ethyl­thio­semi­carbazone

**DOI:** 10.1107/S1600536810018209

**Published:** 2010-05-22

**Authors:** M. R. Vikneswaran, Siang Guan Teoh, Chin Sing Yeap, Hoong-Kun Fun

**Affiliations:** a School of Chemical Sciences, Universiti Sains Malaysia, 11800 USM, Penang, Malaysia; b X-ray Crystallography Unit, School of Physics, Universiti Sains Malaysia, 11800 USM, Penang, Malaysia

## Abstract

The asymmetric unit of title compound, [Fe(C_5_H_5_)(C_9_H_12_N_3_S)], contains two crystallographically independent mol­ecules, *A* and *B*. The two cyclo­penta­dienyl (Cp) rings are parallel to each other in both mol­ecules, forming dihedral angles of 2.3 (3) and 1.0 (3)°, respectively, and adopt an eclipsed conformation. The mean plane of the semicarbazone group is twisted slightly away from the attached Cp ring in both mol­ecules, the dihedral angles between the mean plane and the Cp ring being 15.3 (2) and 10.8 (2)°. The ethyl group in mol­ecule *A* is coplanar with the mean plane of the semicarbazone group [C—N—C—C torsion angle = −175.2 (4)°], whereas it is nearly perpendicular in mol­ecule *B* [C—N—C—C torsion angle = 84.8 (6)°]. In the crystal structure, inter­molecular N—H⋯S hydrogen bonds link the mol­ecules into dimers. These dimers are further linked into chains *via* inter­molecular C—H⋯S hydrogen bonds. The crystal studied was a non-merohedral twin with a refined ratio of the twin components of 0.265 (2):0.735 (2).

## Related literature

For related structures, see: Vikneswaran *et al.* (2009 ▶, 2010 ▶). For the preparation of the title compound, see: Casas *et al.* (2004 ▶). For the stability of the temperature controller used for the data collection, see: Cosier & Glazer (1986 ▶).
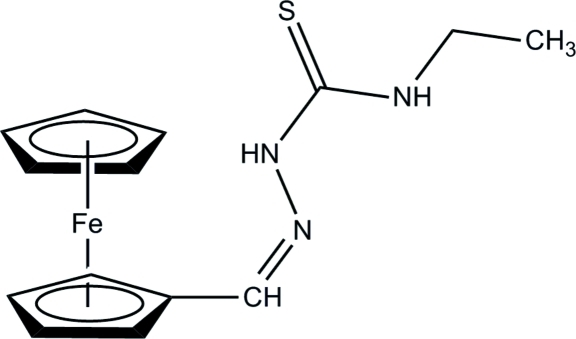


## Experimental

### 

#### Crystal data

[Fe(C_5_H_5_)(C_9_H_12_N_3_S)]
*M* *_r_* = 315.22
Triclinic, 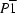

*a* = 7.4432 (3) Å
*b* = 10.6906 (5) Å
*c* = 18.4616 (9) Å
α = 77.975 (3)°
β = 83.807 (3)°
γ = 78.076 (3)°
*V* = 1402.56 (11) Å^3^
*Z* = 4
Mo *K*α radiation
μ = 1.21 mm^−1^
*T* = 100 K
0.29 × 0.16 × 0.09 mm

#### Data collection

Bruker SMART APEXII CCD area-detector diffractometer
Absorption correction: multi-scan (*SADABS*; Bruker, 2009 ▶) *T*_min_ = 0.723, *T*_max_ = 0.901
8184 measured reflections
8184 independent reflections
6947 reflections with *I* > 2σ(*I*)

#### Refinement

*R*[*F* ^2^ > 2σ(*F* ^2^)] = 0.073
*wR*(*F* ^2^) = 0.170
*S* = 1.07
8184 reflections
362 parameters
H atoms treated by a mixture of independent and constrained refinement
Δρ_max_ = 3.94 e Å^−3^
Δρ_min_ = −1.22 e Å^−3^

### 

Data collection: *APEX2* (Bruker, 2009 ▶); cell refinement: *SAINT* (Bruker, 2009 ▶); data reduction: *SAINT*; program(s) used to solve structure: *SHELXTL* (Sheldrick, 2008 ▶); program(s) used to refine structure: *SHELXTL*; molecular graphics: *SHELXTL*; software used to prepare material for publication: *SHELXTL* (Sheldrick, 2008 ▶) and *PLATON* (Spek, 2009 ▶).

## Supplementary Material

Crystal structure: contains datablocks global, I. DOI: 10.1107/S1600536810018209/rz2450sup1.cif

Structure factors: contains datablocks I. DOI: 10.1107/S1600536810018209/rz2450Isup2.hkl

Additional supplementary materials:  crystallographic information; 3D view; checkCIF report

## Figures and Tables

**Table 1:** Hydrogen-bond geometry (Å, °)

*D*—H⋯*A*	*D*—H	H⋯*A*	*D*⋯*A*	*D*—H⋯*A*
N2*A*—H2*NA*⋯S1*A*^i^	0.82 (6)	2.59 (6)	3.387 (4)	164 (5)
N2*B*—H2*NB*⋯S1*B*^ii^	0.89 (9)	2.55 (9)	3.430 (5)	170 (5)
C4*A*—H4*AA*⋯S1*B*^iii^	0.98	2.79	3.715 (4)	157

Symmetry codes: (i) 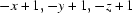; (ii) 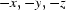; (iii) 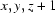.

**Fig. 1.**
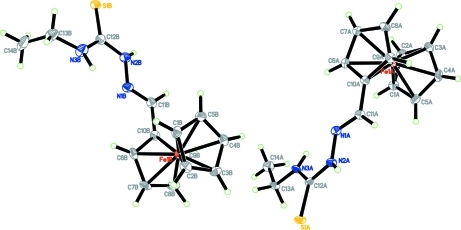
The molecular structure of the title compound, showing 50% probability displacement ellipsoids and the atom-numbering scheme.

**Fig. 2.**
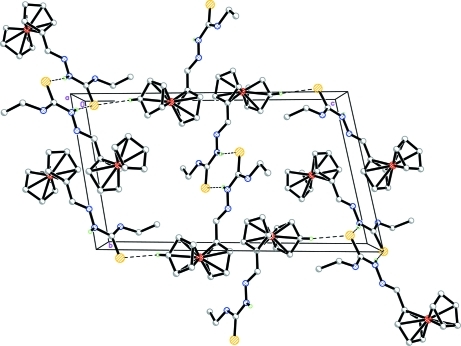
The crystal packing of the title compound, viewed along the a axis, showing the molecules link into 1-D chains. Hydrogen atoms not involved in the hydrogen-bonding (dashed lines) are omitted for clarity.

## supplementary crystallographic information

### Comment

As a continuation of our research related to ferrocenyl thiosemicarbazones and its metal complexes, herein we report the crystal structure of formylferrocene 4-ethylthiosemicarbazone.

The asymmetric unit of title compound consists of two crystallographically independent molecules, *A* and *B *(Fig. 1). The geometric parameter are comparable to those observed in its closely related structures (Vikneswaran *et al.*, 2009, 2010). The Cp rings of each ferrocene residue are parallel, with dihedral angles of Cp1/Cp2 [C1A–C5A/C6A–C10A] = 2.3 (3)° and Cp3/Cp4 [C1B–C5B/C6B–C10B] = 1.0 (3)°. The Cp rings in both molecules adopt an eclipsed conformation [average torsion angles for C–Cg–Cg–C of 5.89 and 6.14°]. The mean plane of the semicarbazone group is slightly twisted away from the attached Cp rings in both molecules, the dihedral angles between the mean plane and the Cp ring being 15.3 (2) and 10.8 (2)° respectively. The ethyl group in molecule *A* is coplanar with the mean plane of semicarbazone group [torsion angle of C12A–N3A–C13A–C14B = -175.2 (4)°] whereas it is nearly perpendicular to the semicarbazone group [torsion angle of C12B–N3B–C13B–C14B = 84.8 (6)°] in molecule *B*.

In the crystal structure, intermolecular N2A–H2NA···S1A and N2B–H2NB···S1B hydrogen bonds link the molecules into dimers. These dimers are linked into one-dimensional chain *via* intermolecular C4A–H4AA···S1B hydrogen bonds (Fig. 2, Table 1).

### Experimental

Formylferrocene 4-ethylthiosemicarbazone was prepared as described by Casas *et al.* (2004). The single crystals were grown from a CH_2_Cl_2_/n-CH_6_H_14_ (1:1 *v*/*v*) solution at room temperature in the dark.

### Refinement

N bound H-atoms were located from difference Fourier map and refined freely. The rest of H-atoms were placed in calculated positions, with C–H = 0.93–0.98 Å and refined using a riding model, with *U*_iso_(H) = 1.2 or 1.5*U*_eq_(C). Rotating-group model were applied for methyl group. The highest residual density peak is located 0.88 Å from atom Fe1B and the deepest hole is located 1.32 Å from atom C12B. The crystal studied is a non-merohedral twin with the refined ratio of twin components of 0.265 (2):0.735 (2).

### Crystal data

**Table:**

[Fe(C_5_H_5_)(C_9_H_12_N_3_S)]	*Z* = 4
*M**_r_* = 315.22	*F*(000) = 656
Triclinic, *P*1	*D*_x_ = 1.493 Mg m^−^^3^
Hall symbol: -P 1	Mo *K*α radiation, λ = 0.71073 Å
*a* = 7.4432 (3) Å	Cell parameters from 9974 reflections
*b* = 10.6906 (5) Å	θ = 2.3–30.0°
*c* = 18.4616 (9) Å	µ = 1.21 mm^−^^1^
α = 77.975 (3)°	*T* = 100 K
β = 83.807 (3)°	Block, brown
γ = 78.076 (3)°	0.29 × 0.16 × 0.09 mm
*V* = 1402.56 (11) Å^3^	

### Data collection

**Table:**

Bruker SMART APEXII CCD area-detector diffractometer	8184 independent reflections
Radiation source: fine-focus sealed tube	6947 reflections with *I* > 2σ(*I*)
graphite	*R*_int_ = 0.0000
φ and ω scans	θ_max_ = 30.1°, θ_min_ = 1.1°
Absorption correction: multi-scan (*SADABS*; Bruker, 2009)	*h* = −10→10
*T*_min_ = 0.723, *T*_max_ = 0.901	*k* = −14→15
8184 measured reflections	*l* = −11→25

### Refinement

**Table:**

Refinement on *F*^2^	Primary atom site location: structure-invariant direct methods
Least-squares matrix: full	Secondary atom site location: difference Fourier map
*R*[*F*^2^ > 2σ(*F*^2^)] = 0.073	Hydrogen site location: inferred from neighbouring sites
*wR*(*F*^2^) = 0.170	H atoms treated by a mixture of independent and constrained refinement
*S* = 1.07	*w* = 1/[σ^2^(*F*_o_^2^) + (0.0564*P*)^2^ + 6.6396*P*] where *P* = (*F*_o_^2^ + 2*F*_c_^2^)/3
8184 reflections	(Δ/σ)_max_ < 0.001
362 parameters	Δρ_max_ = 3.94 e Å^−^^3^
0 restraints	Δρ_min_ = −1.22 e Å^−^^3^

### Special details

**Table:**

Experimental. The crystal was placed in the cold stream of an Oxford Cryosystems Cobra open-flow nitrogen cryostat (Cosier & Glazer, 1986) operating at 100.0 (1) K.
Geometry. All esds (except the esd in the dihedral angle between two l.s. planes) are estimated using the full covariance matrix. The cell esds are taken into account individually in the estimation of esds in distances, angles and torsion angles; correlations between esds in cell parameters are only used when they are defined by crystal symmetry. An approximate (isotropic) treatment of cell esds is used for estimating esds involving l.s. planes.
Refinement. Refinement of *F*^2^ against ALL reflections. The weighted *R*-factor wR and goodness of fit *S* are based on *F*^2^, conventional *R*-factors *R* are based on *F*, with *F* set to zero for negative *F*^2^. The threshold expression of *F*^2^ > σ(*F*^2^) is used only for calculating *R*-factors(gt) etc. and is not relevant to the choice of reflections for refinement. *R*-factors based on *F*^2^ are statistically about twice as large as those based on *F*, and *R*- factors based on ALL data will be even larger.

### Fractional atomic coordinates and isotropic or equivalent isotropic displacement parameters (Å^2^)

**Table:**

	*x*	*y*	*z*	*U*_iso_*/*U*_eq_	
Fe1A	0.13896 (8)	−0.05245 (6)	0.63951 (3)	0.01124 (13)	
S1A	0.29669 (15)	0.61964 (10)	0.42316 (6)	0.0175 (2)	
N1A	0.2762 (5)	0.2535 (3)	0.5056 (2)	0.0145 (7)	
N2A	0.3209 (5)	0.3757 (3)	0.4940 (2)	0.0150 (7)	
N3A	0.0898 (5)	0.4487 (3)	0.4141 (2)	0.0140 (7)	
C1A	−0.1290 (6)	0.0406 (4)	0.6505 (2)	0.0171 (8)	
H1AA	−0.2002	0.0985	0.6108	0.021*	
C2A	−0.1151 (6)	−0.0966 (4)	0.6707 (2)	0.0174 (8)	
H2AA	−0.1754	−0.1498	0.6474	0.021*	
C3A	0.0008 (6)	−0.1433 (4)	0.7305 (2)	0.0201 (9)	
H3AA	0.0348	−0.2343	0.7555	0.024*	
C4A	0.0590 (7)	−0.0348 (5)	0.7480 (2)	0.0211 (9)	
H4AA	0.1403	−0.0376	0.7869	0.025*	
C5A	−0.0221 (6)	0.0788 (5)	0.6976 (3)	0.0203 (9)	
H5AA	−0.0052	0.1679	0.6959	0.024*	
C6A	0.2245 (5)	−0.0166 (4)	0.5289 (2)	0.0137 (7)	
H6AA	0.1491	0.0332	0.4883	0.016*	
C7A	0.2465 (6)	−0.1531 (4)	0.5565 (2)	0.0151 (8)	
H7AA	0.1878	−0.2136	0.5384	0.018*	
C8A	0.3655 (6)	−0.1867 (4)	0.6163 (2)	0.0168 (8)	
H8AA	0.4035	−0.2740	0.6458	0.020*	
C9A	0.4196 (5)	−0.0705 (4)	0.6252 (2)	0.0154 (8)	
H9AA	0.5011	−0.0638	0.6620	0.018*	
C10A	0.3323 (5)	0.0348 (4)	0.5711 (2)	0.0123 (7)	
C11A	0.3566 (5)	0.1695 (4)	0.5581 (2)	0.0146 (8)	
H11A	0.4304	0.1947	0.5879	0.018*	
C12A	0.2306 (5)	0.4739 (4)	0.4442 (2)	0.0125 (7)	
C13A	−0.0243 (6)	0.5431 (4)	0.3617 (2)	0.0151 (8)	
H13A	0.0495	0.5685	0.3164	0.018*	
H13B	−0.0741	0.6203	0.3825	0.018*	
C14A	−0.1810 (6)	0.4851 (5)	0.3440 (3)	0.0223 (9)	
H14A	−0.2522	0.5466	0.3073	0.033*	
H14B	−0.2583	0.4652	0.3883	0.033*	
H14C	−0.1314	0.4067	0.3253	0.033*	
Fe1B	0.30485 (8)	0.46654 (6)	0.14382 (3)	0.01438 (14)	
S1B	0.24370 (18)	−0.06556 (11)	−0.06872 (7)	0.0224 (2)	
N1B	0.2135 (5)	0.2488 (4)	0.0132 (2)	0.0195 (8)	
N2B	0.1830 (6)	0.1303 (4)	0.0031 (2)	0.0223 (8)	
N3B	0.4293 (6)	0.1273 (4)	−0.0817 (2)	0.0252 (9)	
C1B	0.5691 (6)	0.3613 (5)	0.1531 (2)	0.0197 (9)	
H1BA	0.6511	0.3301	0.1127	0.024*	
C2B	0.5528 (6)	0.4837 (4)	0.1738 (3)	0.0189 (9)	
H2BA	0.6230	0.5516	0.1507	0.023*	
C3B	0.4170 (6)	0.4906 (4)	0.2346 (3)	0.0187 (8)	
H3BA	0.3781	0.5635	0.2609	0.022*	
C4B	0.3493 (6)	0.3726 (4)	0.2500 (2)	0.0190 (8)	
H4BA	0.2539	0.3502	0.2887	0.023*	
C5B	0.4432 (7)	0.2922 (4)	0.2007 (3)	0.0209 (9)	
H5BA	0.4231	0.2052	0.1987	0.025*	
C6B	0.2535 (6)	0.5058 (5)	0.0337 (2)	0.0195 (9)	
H6BA	0.3413	0.4832	−0.0072	0.023*	
C7B	0.2245 (7)	0.6241 (4)	0.0616 (3)	0.0221 (9)	
H7BA	0.2895	0.6967	0.0433	0.026*	
C8B	0.0860 (6)	0.6177 (4)	0.1209 (3)	0.0226 (9)	
H8BA	0.0391	0.6850	0.1506	0.027*	
C9B	0.0287 (6)	0.4950 (5)	0.1302 (3)	0.0205 (9)	
H9BA	−0.0648	0.4638	0.1672	0.025*	
C10B	0.1320 (6)	0.4262 (4)	0.0763 (2)	0.0171 (8)	
C11B	0.1143 (6)	0.2982 (4)	0.0654 (3)	0.0193 (9)	
H11B	0.0320	0.2527	0.0960	0.023*	
C12B	0.2896 (7)	0.0721 (5)	−0.0494 (3)	0.0204 (9)	
C13B	0.5572 (7)	0.0852 (5)	−0.1416 (3)	0.0284 (11)	
H13C	0.5809	−0.0091	−0.1340	0.034*	
H13D	0.6731	0.1122	−0.1398	0.034*	
C14B	0.4841 (9)	0.1411 (6)	−0.2179 (3)	0.0374 (13)	
H14D	0.5742	0.1130	−0.2553	0.056*	
H14E	0.4593	0.2345	−0.2256	0.056*	
H14F	0.3727	0.1109	−0.2208	0.056*	
H2NA	0.416 (8)	0.390 (5)	0.507 (3)	0.016 (13)*	
H2NB	0.073 (12)	0.109 (8)	0.015 (5)	0.07 (3)*	
H3NB	0.458 (11)	0.186 (7)	−0.053 (4)	0.05 (2)*	
H3NA	0.046 (8)	0.384 (6)	0.437 (3)	0.026 (15)*	

### Atomic displacement parameters (Å^2^)

**Table:**

	*U*^11^	*U*^22^	*U*^33^	*U*^12^	*U*^13^	*U*^23^
Fe1A	0.0099 (3)	0.0130 (3)	0.0111 (3)	−0.0036 (2)	−0.0009 (2)	−0.0015 (2)
S1A	0.0198 (5)	0.0117 (5)	0.0231 (5)	−0.0066 (4)	−0.0057 (4)	−0.0027 (4)
N1A	0.0116 (15)	0.0160 (17)	0.0175 (17)	−0.0050 (13)	−0.0004 (13)	−0.0046 (13)
N2A	0.0147 (16)	0.0125 (16)	0.0189 (17)	−0.0042 (13)	−0.0027 (13)	−0.0028 (13)
N3A	0.0161 (16)	0.0100 (15)	0.0162 (17)	−0.0061 (13)	−0.0021 (13)	0.0009 (13)
C1A	0.0122 (17)	0.018 (2)	0.020 (2)	−0.0018 (15)	0.0021 (15)	−0.0048 (16)
C2A	0.0162 (18)	0.018 (2)	0.021 (2)	−0.0094 (15)	0.0027 (16)	−0.0045 (16)
C3A	0.025 (2)	0.018 (2)	0.016 (2)	−0.0071 (17)	0.0021 (17)	0.0007 (16)
C4A	0.025 (2)	0.028 (2)	0.0121 (19)	−0.0105 (18)	0.0030 (16)	−0.0041 (17)
C5A	0.026 (2)	0.020 (2)	0.018 (2)	−0.0067 (17)	0.0034 (17)	−0.0086 (17)
C6A	0.0102 (17)	0.0179 (19)	0.0135 (18)	−0.0039 (14)	0.0000 (14)	−0.0032 (15)
C7A	0.0132 (17)	0.0169 (19)	0.0153 (19)	−0.0026 (14)	−0.0007 (14)	−0.0037 (15)
C8A	0.0136 (18)	0.0140 (19)	0.021 (2)	0.0005 (14)	−0.0020 (15)	−0.0006 (15)
C9A	0.0098 (16)	0.018 (2)	0.0181 (19)	−0.0022 (14)	−0.0029 (14)	−0.0026 (15)
C10A	0.0086 (16)	0.0145 (18)	0.0136 (18)	−0.0033 (14)	0.0015 (13)	−0.0023 (14)
C11A	0.0114 (17)	0.0159 (19)	0.0175 (19)	−0.0048 (14)	−0.0015 (14)	−0.0031 (15)
C12A	0.0135 (17)	0.0125 (18)	0.0133 (18)	−0.0047 (14)	0.0006 (14)	−0.0053 (14)
C13A	0.0137 (17)	0.0115 (18)	0.019 (2)	−0.0021 (14)	−0.0043 (15)	−0.0001 (15)
C14A	0.018 (2)	0.025 (2)	0.026 (2)	−0.0062 (17)	−0.0062 (17)	−0.0051 (18)
Fe1B	0.0136 (3)	0.0156 (3)	0.0144 (3)	−0.0045 (2)	−0.0029 (2)	−0.0013 (2)
S1B	0.0350 (6)	0.0162 (5)	0.0199 (5)	−0.0113 (4)	−0.0027 (4)	−0.0051 (4)
N1B	0.0208 (18)	0.0182 (18)	0.0225 (19)	−0.0076 (14)	−0.0043 (15)	−0.0056 (15)
N2B	0.029 (2)	0.0195 (19)	0.023 (2)	−0.0120 (16)	−0.0028 (16)	−0.0053 (15)
N3B	0.0226 (19)	0.031 (2)	0.029 (2)	−0.0131 (17)	−0.0025 (16)	−0.0129 (18)
C1B	0.019 (2)	0.024 (2)	0.016 (2)	−0.0011 (17)	−0.0039 (16)	−0.0049 (17)
C2B	0.0118 (18)	0.021 (2)	0.024 (2)	−0.0075 (16)	−0.0022 (15)	−0.0016 (17)
C3B	0.0182 (19)	0.017 (2)	0.023 (2)	−0.0021 (16)	−0.0045 (16)	−0.0076 (16)
C4B	0.0179 (19)	0.021 (2)	0.017 (2)	−0.0045 (16)	−0.0027 (16)	−0.0009 (16)
C5B	0.026 (2)	0.016 (2)	0.021 (2)	−0.0027 (17)	−0.0045 (17)	−0.0033 (16)
C6B	0.021 (2)	0.023 (2)	0.0134 (19)	−0.0066 (17)	−0.0042 (16)	0.0020 (16)
C7B	0.031 (2)	0.0121 (19)	0.022 (2)	−0.0031 (17)	−0.0130 (19)	0.0039 (16)
C8B	0.023 (2)	0.015 (2)	0.029 (2)	0.0032 (17)	−0.0095 (18)	−0.0050 (17)
C9B	0.0165 (19)	0.024 (2)	0.021 (2)	−0.0053 (17)	−0.0042 (16)	−0.0028 (17)
C10B	0.0169 (19)	0.018 (2)	0.0171 (19)	−0.0042 (15)	−0.0068 (15)	−0.0019 (16)
C11B	0.019 (2)	0.022 (2)	0.020 (2)	−0.0095 (17)	−0.0035 (16)	−0.0021 (17)
C12B	0.025 (2)	0.020 (2)	0.018 (2)	−0.0074 (17)	−0.0062 (17)	−0.0029 (16)
C13B	0.023 (2)	0.030 (3)	0.035 (3)	−0.0052 (19)	0.003 (2)	−0.014 (2)
C14B	0.041 (3)	0.046 (3)	0.033 (3)	−0.018 (3)	0.014 (2)	−0.024 (3)

### Geometric parameters (Å, °)

**Table:**

Fe1A—C2A	2.037 (4)	Fe1B—C3B	2.036 (4)
Fe1A—C3A	2.046 (4)	Fe1B—C4B	2.036 (4)
Fe1A—C1A	2.046 (4)	Fe1B—C2B	2.037 (4)
Fe1A—C8A	2.046 (4)	Fe1B—C10B	2.048 (4)
Fe1A—C9A	2.051 (4)	Fe1B—C9B	2.050 (4)
Fe1A—C10A	2.051 (4)	Fe1B—C6B	2.051 (4)
Fe1A—C5A	2.052 (5)	Fe1B—C7B	2.053 (4)
Fe1A—C7A	2.052 (4)	Fe1B—C8B	2.054 (4)
Fe1A—C6A	2.053 (4)	Fe1B—C1B	2.060 (4)
Fe1A—C4A	2.063 (4)	Fe1B—C5B	2.064 (5)
S1A—C12A	1.687 (4)	S1B—C12B	1.691 (5)
N1A—C11A	1.283 (5)	N1B—C11B	1.285 (6)
N1A—N2A	1.383 (5)	N1B—N2B	1.384 (5)
N2A—C12A	1.356 (5)	N2B—C12B	1.357 (7)
N2A—H2NA	0.82 (6)	N2B—H2NB	0.89 (9)
N3A—C12A	1.335 (5)	N3B—C12B	1.326 (6)
N3A—C13A	1.447 (5)	N3B—C13B	1.461 (7)
N3A—H3NA	0.84 (6)	N3B—H3NB	0.97 (8)
C1A—C5A	1.410 (6)	C1B—C2B	1.417 (7)
C1A—C2A	1.421 (6)	C1B—C5B	1.426 (6)
C1A—H1AA	0.9800	C1B—H1BA	0.9800
C2A—C3A	1.418 (6)	C2B—C3B	1.431 (6)
C2A—H2AA	0.9800	C2B—H2BA	0.9800
C3A—C4A	1.425 (7)	C3B—C4B	1.418 (6)
C3A—H3AA	0.9800	C3B—H3BA	0.9800
C4A—C5A	1.433 (7)	C4B—C5B	1.414 (7)
C4A—H4AA	0.9800	C4B—H4BA	0.9800
C5A—H5AA	0.9800	C5B—H5BA	0.9800
C6A—C7A	1.424 (6)	C6B—C10B	1.431 (6)
C6A—C10A	1.430 (5)	C6B—C7B	1.431 (7)
C6A—H6AA	0.9800	C6B—H6BA	0.9800
C7A—C8A	1.429 (6)	C7B—C8B	1.421 (7)
C7A—H7AA	0.9800	C7B—H7BA	0.9800
C8A—C9A	1.428 (6)	C8B—C9B	1.433 (6)
C8A—H8AA	0.9800	C8B—H8BA	0.9800
C9A—C10A	1.434 (6)	C9B—C10B	1.424 (7)
C9A—H9AA	0.9800	C9B—H9BA	0.9800
C10A—C11A	1.456 (6)	C10B—C11B	1.459 (6)
C11A—H11A	0.9300	C11B—H11B	0.9300
C13A—C14A	1.523 (6)	C13B—C14B	1.522 (8)
C13A—H13A	0.9700	C13B—H13C	0.9700
C13A—H13B	0.9700	C13B—H13D	0.9700
C14A—H14A	0.9600	C14B—H14D	0.9600
C14A—H14B	0.9600	C14B—H14E	0.9600
C14A—H14C	0.9600	C14B—H14F	0.9600
			
C2A—Fe1A—C3A	40.63 (18)	C3B—Fe1B—C4B	40.76 (18)
C2A—Fe1A—C1A	40.72 (17)	C3B—Fe1B—C2B	41.13 (18)
C3A—Fe1A—C1A	68.45 (18)	C4B—Fe1B—C2B	68.65 (18)
C2A—Fe1A—C8A	123.50 (18)	C3B—Fe1B—C10B	162.68 (18)
C3A—Fe1A—C8A	106.06 (18)	C4B—Fe1B—C10B	125.67 (17)
C1A—Fe1A—C8A	161.08 (18)	C2B—Fe1B—C10B	154.96 (19)
C2A—Fe1A—C9A	161.10 (17)	C3B—Fe1B—C9B	125.40 (19)
C3A—Fe1A—C9A	124.71 (18)	C4B—Fe1B—C9B	108.12 (18)
C1A—Fe1A—C9A	157.02 (18)	C2B—Fe1B—C9B	162.68 (19)
C8A—Fe1A—C9A	40.79 (17)	C10B—Fe1B—C9B	40.67 (19)
C2A—Fe1A—C10A	155.75 (17)	C3B—Fe1B—C6B	155.00 (18)
C3A—Fe1A—C10A	162.98 (17)	C4B—Fe1B—C6B	162.63 (19)
C1A—Fe1A—C10A	121.71 (17)	C2B—Fe1B—C6B	119.64 (18)
C8A—Fe1A—C10A	68.66 (16)	C10B—Fe1B—C6B	40.86 (17)
C9A—Fe1A—C10A	40.93 (16)	C9B—Fe1B—C6B	68.61 (18)
C2A—Fe1A—C5A	68.06 (18)	C3B—Fe1B—C7B	120.00 (18)
C3A—Fe1A—C5A	68.28 (19)	C4B—Fe1B—C7B	155.5 (2)
C1A—Fe1A—C5A	40.26 (18)	C2B—Fe1B—C7B	106.91 (18)
C8A—Fe1A—C5A	156.34 (18)	C10B—Fe1B—C7B	68.64 (18)
C9A—Fe1A—C5A	122.29 (18)	C9B—Fe1B—C7B	68.55 (19)
C10A—Fe1A—C5A	109.67 (17)	C6B—Fe1B—C7B	40.81 (19)
C2A—Fe1A—C7A	106.16 (17)	C3B—Fe1B—C8B	107.44 (19)
C3A—Fe1A—C7A	119.01 (18)	C4B—Fe1B—C8B	121.0 (2)
C1A—Fe1A—C7A	124.63 (17)	C2B—Fe1B—C8B	125.02 (19)
C8A—Fe1A—C7A	40.82 (16)	C10B—Fe1B—C8B	68.52 (18)
C9A—Fe1A—C7A	68.53 (17)	C9B—Fe1B—C8B	40.87 (18)
C10A—Fe1A—C7A	68.29 (16)	C6B—Fe1B—C8B	68.4 (2)
C5A—Fe1A—C7A	162.30 (18)	C7B—Fe1B—C8B	40.5 (2)
C2A—Fe1A—C6A	119.68 (17)	C3B—Fe1B—C1B	68.50 (18)
C3A—Fe1A—C6A	154.10 (18)	C4B—Fe1B—C1B	68.19 (18)
C1A—Fe1A—C6A	107.78 (17)	C2B—Fe1B—C1B	40.47 (18)
C8A—Fe1A—C6A	68.80 (17)	C10B—Fe1B—C1B	120.59 (18)
C9A—Fe1A—C6A	68.88 (16)	C9B—Fe1B—C1B	155.71 (19)
C10A—Fe1A—C6A	40.79 (15)	C6B—Fe1B—C1B	107.35 (19)
C5A—Fe1A—C6A	126.39 (18)	C7B—Fe1B—C1B	125.0 (2)
C7A—Fe1A—C6A	40.58 (16)	C8B—Fe1B—C1B	161.93 (19)
C2A—Fe1A—C4A	68.27 (18)	C3B—Fe1B—C5B	68.25 (18)
C3A—Fe1A—C4A	40.57 (18)	C4B—Fe1B—C5B	40.33 (19)
C1A—Fe1A—C4A	68.32 (19)	C2B—Fe1B—C5B	68.20 (18)
C8A—Fe1A—C4A	120.07 (19)	C10B—Fe1B—C5B	108.19 (18)
C9A—Fe1A—C4A	108.26 (18)	C9B—Fe1B—C5B	121.11 (19)
C10A—Fe1A—C4A	126.87 (18)	C6B—Fe1B—C5B	125.56 (19)
C5A—Fe1A—C4A	40.75 (19)	C7B—Fe1B—C5B	162.4 (2)
C7A—Fe1A—C4A	154.51 (18)	C8B—Fe1B—C5B	156.1 (2)
C6A—Fe1A—C4A	163.95 (18)	C1B—Fe1B—C5B	40.47 (18)
C11A—N1A—N2A	115.2 (3)	C11B—N1B—N2B	116.6 (4)
C12A—N2A—N1A	119.4 (3)	C12B—N2B—N1B	118.6 (4)
C12A—N2A—H2NA	115 (4)	C12B—N2B—H2NB	116 (5)
N1A—N2A—H2NA	124 (4)	N1B—N2B—H2NB	121 (6)
C12A—N3A—C13A	124.1 (3)	C12B—N3B—C13B	126.0 (4)
C12A—N3A—H3NA	116 (4)	C12B—N3B—H3NB	111 (5)
C13A—N3A—H3NA	117 (4)	C13B—N3B—H3NB	121 (5)
C5A—C1A—C2A	107.9 (4)	C2B—C1B—C5B	107.9 (4)
C5A—C1A—Fe1A	70.1 (3)	C2B—C1B—Fe1B	68.9 (2)
C2A—C1A—Fe1A	69.3 (2)	C5B—C1B—Fe1B	69.9 (3)
C5A—C1A—H1AA	126.1	C2B—C1B—H1BA	126.0
C2A—C1A—H1AA	126.1	C5B—C1B—H1BA	126.0
Fe1A—C1A—H1AA	126.1	Fe1B—C1B—H1BA	126.0
C3A—C2A—C1A	108.3 (4)	C1B—C2B—C3B	108.1 (4)
C3A—C2A—Fe1A	70.0 (2)	C1B—C2B—Fe1B	70.6 (2)
C1A—C2A—Fe1A	70.0 (2)	C3B—C2B—Fe1B	69.4 (2)
C3A—C2A—H2AA	125.8	C1B—C2B—H2BA	126.0
C1A—C2A—H2AA	125.8	C3B—C2B—H2BA	126.0
Fe1A—C2A—H2AA	125.8	Fe1B—C2B—H2BA	126.0
C2A—C3A—C4A	108.1 (4)	C4B—C3B—C2B	107.5 (4)
C2A—C3A—Fe1A	69.4 (2)	C4B—C3B—Fe1B	69.6 (2)
C4A—C3A—Fe1A	70.4 (2)	C2B—C3B—Fe1B	69.5 (2)
C2A—C3A—H3AA	125.9	C4B—C3B—H3BA	126.3
C4A—C3A—H3AA	125.9	C2B—C3B—H3BA	126.3
Fe1A—C3A—H3AA	125.9	Fe1B—C3B—H3BA	126.3
C3A—C4A—C5A	107.2 (4)	C5B—C4B—C3B	108.6 (4)
C3A—C4A—Fe1A	69.1 (3)	C5B—C4B—Fe1B	70.9 (3)
C5A—C4A—Fe1A	69.2 (3)	C3B—C4B—Fe1B	69.6 (3)
C3A—C4A—H4AA	126.4	C5B—C4B—H4BA	125.7
C5A—C4A—H4AA	126.4	C3B—C4B—H4BA	125.7
Fe1A—C4A—H4AA	126.4	Fe1B—C4B—H4BA	125.7
C1A—C5A—C4A	108.5 (4)	C4B—C5B—C1B	107.9 (4)
C1A—C5A—Fe1A	69.6 (3)	C4B—C5B—Fe1B	68.8 (3)
C4A—C5A—Fe1A	70.0 (3)	C1B—C5B—Fe1B	69.6 (3)
C1A—C5A—H5AA	125.8	C4B—C5B—H5BA	126.1
C4A—C5A—H5AA	125.8	C1B—C5B—H5BA	126.1
Fe1A—C5A—H5AA	125.8	Fe1B—C5B—H5BA	126.1
C7A—C6A—C10A	107.6 (3)	C10B—C6B—C7B	107.8 (4)
C7A—C6A—Fe1A	69.7 (2)	C10B—C6B—Fe1B	69.5 (2)
C10A—C6A—Fe1A	69.5 (2)	C7B—C6B—Fe1B	69.7 (2)
C7A—C6A—H6AA	126.2	C10B—C6B—H6BA	126.1
C10A—C6A—H6AA	126.2	C7B—C6B—H6BA	126.1
Fe1A—C6A—H6AA	126.2	Fe1B—C6B—H6BA	126.1
C6A—C7A—C8A	108.5 (4)	C8B—C7B—C6B	108.1 (4)
C6A—C7A—Fe1A	69.7 (2)	C8B—C7B—Fe1B	69.8 (3)
C8A—C7A—Fe1A	69.4 (3)	C6B—C7B—Fe1B	69.5 (2)
C6A—C7A—H7AA	125.7	C8B—C7B—H7BA	126.0
C8A—C7A—H7AA	125.7	C6B—C7B—H7BA	126.0
Fe1A—C7A—H7AA	125.7	Fe1B—C7B—H7BA	126.0
C9A—C8A—C7A	107.9 (4)	C7B—C8B—C9B	108.1 (4)
C9A—C8A—Fe1A	69.8 (2)	C7B—C8B—Fe1B	69.7 (3)
C7A—C8A—Fe1A	69.8 (2)	C9B—C8B—Fe1B	69.4 (3)
C9A—C8A—H8AA	126.0	C7B—C8B—H8BA	125.9
C7A—C8A—H8AA	126.0	C9B—C8B—H8BA	125.9
Fe1A—C8A—H8AA	126.0	Fe1B—C8B—H8BA	125.9
C8A—C9A—C10A	107.7 (3)	C10B—C9B—C8B	107.9 (4)
C8A—C9A—Fe1A	69.4 (2)	C10B—C9B—Fe1B	69.6 (2)
C10A—C9A—Fe1A	69.6 (2)	C8B—C9B—Fe1B	69.7 (2)
C8A—C9A—H9AA	126.1	C10B—C9B—H9BA	126.1
C10A—C9A—H9AA	126.1	C8B—C9B—H9BA	126.1
Fe1A—C9A—H9AA	126.1	Fe1B—C9B—H9BA	126.1
C6A—C10A—C9A	108.2 (4)	C9B—C10B—C6B	108.1 (4)
C6A—C10A—C11A	125.5 (4)	C9B—C10B—C11B	126.0 (4)
C9A—C10A—C11A	126.2 (4)	C6B—C10B—C11B	125.9 (4)
C6A—C10A—Fe1A	69.7 (2)	C9B—C10B—Fe1B	69.8 (2)
C9A—C10A—Fe1A	69.5 (2)	C6B—C10B—Fe1B	69.7 (2)
C11A—C10A—Fe1A	129.0 (3)	C11B—C10B—Fe1B	127.2 (3)
N1A—C11A—C10A	120.0 (4)	N1B—C11B—C10B	119.4 (4)
N1A—C11A—H11A	120.0	N1B—C11B—H11B	120.3
C10A—C11A—H11A	120.0	C10B—C11B—H11B	120.3
N3A—C12A—N2A	116.8 (4)	N3B—C12B—N2B	116.3 (4)
N3A—C12A—S1A	123.0 (3)	N3B—C12B—S1B	123.8 (4)
N2A—C12A—S1A	120.1 (3)	N2B—C12B—S1B	119.9 (4)
N3A—C13A—C14A	110.0 (3)	N3B—C13B—C14B	112.6 (4)
N3A—C13A—H13A	109.7	N3B—C13B—H13C	109.1
C14A—C13A—H13A	109.7	C14B—C13B—H13C	109.1
N3A—C13A—H13B	109.7	N3B—C13B—H13D	109.1
C14A—C13A—H13B	109.7	C14B—C13B—H13D	109.1
H13A—C13A—H13B	108.2	H13C—C13B—H13D	107.8
C13A—C14A—H14A	109.5	C13B—C14B—H14D	109.5
C13A—C14A—H14B	109.5	C13B—C14B—H14E	109.5
H14A—C14A—H14B	109.5	H14D—C14B—H14E	109.5
C13A—C14A—H14C	109.5	C13B—C14B—H14F	109.5
H14A—C14A—H14C	109.5	H14D—C14B—H14F	109.5
H14B—C14A—H14C	109.5	H14E—C14B—H14F	109.5
			
C11A—N1A—N2A—C12A	−174.2 (4)	C11B—N1B—N2B—C12B	−177.4 (4)
C2A—Fe1A—C1A—C5A	119.1 (4)	C3B—Fe1B—C1B—C2B	−38.2 (3)
C3A—Fe1A—C1A—C5A	81.4 (3)	C4B—Fe1B—C1B—C2B	−82.2 (3)
C8A—Fe1A—C1A—C5A	157.8 (5)	C10B—Fe1B—C1B—C2B	158.2 (3)
C9A—Fe1A—C1A—C5A	−48.1 (6)	C9B—Fe1B—C1B—C2B	−168.1 (4)
C10A—Fe1A—C1A—C5A	−83.3 (3)	C6B—Fe1B—C1B—C2B	115.6 (3)
C7A—Fe1A—C1A—C5A	−167.4 (3)	C7B—Fe1B—C1B—C2B	74.1 (3)
C6A—Fe1A—C1A—C5A	−125.8 (3)	C8B—Fe1B—C1B—C2B	42.1 (7)
C4A—Fe1A—C1A—C5A	37.6 (3)	C5B—Fe1B—C1B—C2B	−119.5 (4)
C3A—Fe1A—C1A—C2A	−37.6 (3)	C3B—Fe1B—C1B—C5B	81.3 (3)
C8A—Fe1A—C1A—C2A	38.8 (6)	C4B—Fe1B—C1B—C5B	37.3 (3)
C9A—Fe1A—C1A—C2A	−167.2 (4)	C2B—Fe1B—C1B—C5B	119.5 (4)
C10A—Fe1A—C1A—C2A	157.7 (3)	C10B—Fe1B—C1B—C5B	−82.3 (3)
C5A—Fe1A—C1A—C2A	−119.1 (4)	C9B—Fe1B—C1B—C5B	−48.6 (5)
C7A—Fe1A—C1A—C2A	73.5 (3)	C6B—Fe1B—C1B—C5B	−124.9 (3)
C6A—Fe1A—C1A—C2A	115.1 (3)	C7B—Fe1B—C1B—C5B	−166.4 (3)
C4A—Fe1A—C1A—C2A	−81.4 (3)	C8B—Fe1B—C1B—C5B	161.6 (6)
C5A—C1A—C2A—C3A	0.0 (5)	C5B—C1B—C2B—C3B	0.3 (5)
Fe1A—C1A—C2A—C3A	59.7 (3)	Fe1B—C1B—C2B—C3B	59.5 (3)
C5A—C1A—C2A—Fe1A	−59.7 (3)	C5B—C1B—C2B—Fe1B	−59.3 (3)
C1A—Fe1A—C2A—C3A	−119.3 (4)	C3B—Fe1B—C2B—C1B	118.9 (4)
C8A—Fe1A—C2A—C3A	74.8 (3)	C4B—Fe1B—C2B—C1B	81.0 (3)
C9A—Fe1A—C2A—C3A	45.2 (7)	C10B—Fe1B—C2B—C1B	−49.0 (5)
C10A—Fe1A—C2A—C3A	−171.2 (4)	C9B—Fe1B—C2B—C1B	163.4 (6)
C5A—Fe1A—C2A—C3A	−81.8 (3)	C6B—Fe1B—C2B—C1B	−82.1 (3)
C7A—Fe1A—C2A—C3A	116.0 (3)	C7B—Fe1B—C2B—C1B	−124.6 (3)
C6A—Fe1A—C2A—C3A	157.8 (3)	C8B—Fe1B—C2B—C1B	−165.3 (3)
C4A—Fe1A—C2A—C3A	−37.7 (3)	C5B—Fe1B—C2B—C1B	37.5 (3)
C3A—Fe1A—C2A—C1A	119.3 (4)	C4B—Fe1B—C2B—C3B	−37.9 (3)
C8A—Fe1A—C2A—C1A	−165.9 (3)	C10B—Fe1B—C2B—C3B	−167.9 (4)
C9A—Fe1A—C2A—C1A	164.5 (5)	C9B—Fe1B—C2B—C3B	44.5 (7)
C10A—Fe1A—C2A—C1A	−51.9 (5)	C6B—Fe1B—C2B—C3B	159.0 (3)
C5A—Fe1A—C2A—C1A	37.5 (3)	C7B—Fe1B—C2B—C3B	116.5 (3)
C7A—Fe1A—C2A—C1A	−124.8 (3)	C8B—Fe1B—C2B—C3B	75.8 (3)
C6A—Fe1A—C2A—C1A	−82.9 (3)	C1B—Fe1B—C2B—C3B	−118.9 (4)
C4A—Fe1A—C2A—C1A	81.6 (3)	C5B—Fe1B—C2B—C3B	−81.4 (3)
C1A—C2A—C3A—C4A	0.3 (5)	C1B—C2B—C3B—C4B	−0.8 (5)
Fe1A—C2A—C3A—C4A	60.0 (3)	Fe1B—C2B—C3B—C4B	59.5 (3)
C1A—C2A—C3A—Fe1A	−59.7 (3)	C1B—C2B—C3B—Fe1B	−60.3 (3)
C1A—Fe1A—C3A—C2A	37.7 (3)	C2B—Fe1B—C3B—C4B	−118.7 (4)
C8A—Fe1A—C3A—C2A	−123.1 (3)	C10B—Fe1B—C3B—C4B	43.9 (7)
C9A—Fe1A—C3A—C2A	−163.8 (3)	C9B—Fe1B—C3B—C4B	76.1 (3)
C10A—Fe1A—C3A—C2A	167.6 (5)	C6B—Fe1B—C3B—C4B	−166.2 (4)
C5A—Fe1A—C3A—C2A	81.2 (3)	C7B—Fe1B—C3B—C4B	159.9 (3)
C7A—Fe1A—C3A—C2A	−80.9 (3)	C8B—Fe1B—C3B—C4B	117.6 (3)
C6A—Fe1A—C3A—C2A	−48.7 (5)	C1B—Fe1B—C3B—C4B	−81.1 (3)
C4A—Fe1A—C3A—C2A	119.1 (4)	C5B—Fe1B—C3B—C4B	−37.4 (3)
C2A—Fe1A—C3A—C4A	−119.1 (4)	C4B—Fe1B—C3B—C2B	118.7 (4)
C1A—Fe1A—C3A—C4A	−81.4 (3)	C10B—Fe1B—C3B—C2B	162.7 (5)
C8A—Fe1A—C3A—C4A	117.7 (3)	C9B—Fe1B—C3B—C2B	−165.2 (3)
C9A—Fe1A—C3A—C4A	77.1 (3)	C6B—Fe1B—C3B—C2B	−47.4 (5)
C10A—Fe1A—C3A—C4A	48.4 (7)	C7B—Fe1B—C3B—C2B	−81.4 (3)
C5A—Fe1A—C3A—C4A	−37.9 (3)	C8B—Fe1B—C3B—C2B	−123.7 (3)
C7A—Fe1A—C3A—C4A	159.9 (3)	C1B—Fe1B—C3B—C2B	37.6 (3)
C6A—Fe1A—C3A—C4A	−167.9 (4)	C5B—Fe1B—C3B—C2B	81.3 (3)
C2A—C3A—C4A—C5A	−0.4 (5)	C2B—C3B—C4B—C5B	1.0 (5)
Fe1A—C3A—C4A—C5A	58.9 (3)	Fe1B—C3B—C4B—C5B	60.4 (3)
C2A—C3A—C4A—Fe1A	−59.3 (3)	C2B—C3B—C4B—Fe1B	−59.4 (3)
C2A—Fe1A—C4A—C3A	37.8 (3)	C3B—Fe1B—C4B—C5B	−119.3 (4)
C1A—Fe1A—C4A—C3A	81.7 (3)	C2B—Fe1B—C4B—C5B	−81.0 (3)
C8A—Fe1A—C4A—C3A	−79.4 (3)	C10B—Fe1B—C4B—C5B	75.4 (3)
C9A—Fe1A—C4A—C3A	−122.4 (3)	C9B—Fe1B—C4B—C5B	117.0 (3)
C10A—Fe1A—C4A—C3A	−164.1 (3)	C6B—Fe1B—C4B—C5B	40.9 (7)
C5A—Fe1A—C4A—C3A	118.9 (4)	C7B—Fe1B—C4B—C5B	−165.1 (4)
C7A—Fe1A—C4A—C3A	−44.2 (5)	C8B—Fe1B—C4B—C5B	160.0 (3)
C6A—Fe1A—C4A—C3A	160.6 (5)	C1B—Fe1B—C4B—C5B	−37.4 (3)
C2A—Fe1A—C4A—C5A	−81.2 (3)	C2B—Fe1B—C4B—C3B	38.3 (3)
C3A—Fe1A—C4A—C5A	−118.9 (4)	C10B—Fe1B—C4B—C3B	−165.3 (3)
C1A—Fe1A—C4A—C5A	−37.2 (3)	C9B—Fe1B—C4B—C3B	−123.6 (3)
C8A—Fe1A—C4A—C5A	161.7 (3)	C6B—Fe1B—C4B—C3B	160.2 (6)
C9A—Fe1A—C4A—C5A	118.6 (3)	C7B—Fe1B—C4B—C3B	−45.7 (6)
C10A—Fe1A—C4A—C5A	77.0 (3)	C8B—Fe1B—C4B—C3B	−80.7 (3)
C7A—Fe1A—C4A—C5A	−163.1 (4)	C1B—Fe1B—C4B—C3B	81.9 (3)
C6A—Fe1A—C4A—C5A	41.7 (7)	C5B—Fe1B—C4B—C3B	119.3 (4)
C2A—C1A—C5A—C4A	−0.2 (5)	C3B—C4B—C5B—C1B	−0.8 (5)
Fe1A—C1A—C5A—C4A	−59.5 (3)	Fe1B—C4B—C5B—C1B	58.8 (3)
C2A—C1A—C5A—Fe1A	59.2 (3)	C3B—C4B—C5B—Fe1B	−59.6 (3)
C3A—C4A—C5A—C1A	0.4 (5)	C2B—C1B—C5B—C4B	0.3 (5)
Fe1A—C4A—C5A—C1A	59.2 (3)	Fe1B—C1B—C5B—C4B	−58.3 (3)
C3A—C4A—C5A—Fe1A	−58.8 (3)	C2B—C1B—C5B—Fe1B	58.6 (3)
C2A—Fe1A—C5A—C1A	−37.9 (3)	C3B—Fe1B—C5B—C4B	37.8 (3)
C3A—Fe1A—C5A—C1A	−81.9 (3)	C2B—Fe1B—C5B—C4B	82.3 (3)
C8A—Fe1A—C5A—C1A	−162.2 (4)	C10B—Fe1B—C5B—C4B	−124.1 (3)
C9A—Fe1A—C5A—C1A	159.9 (3)	C9B—Fe1B—C5B—C4B	−81.4 (3)
C10A—Fe1A—C5A—C1A	116.2 (3)	C6B—Fe1B—C5B—C4B	−166.1 (3)
C7A—Fe1A—C5A—C1A	36.1 (7)	C7B—Fe1B—C5B—C4B	159.3 (5)
C6A—Fe1A—C5A—C1A	73.5 (3)	C8B—Fe1B—C5B—C4B	−46.3 (6)
C4A—Fe1A—C5A—C1A	−119.7 (4)	C1B—Fe1B—C5B—C4B	119.7 (4)
C2A—Fe1A—C5A—C4A	81.7 (3)	C3B—Fe1B—C5B—C1B	−81.9 (3)
C3A—Fe1A—C5A—C4A	37.8 (3)	C4B—Fe1B—C5B—C1B	−119.7 (4)
C1A—Fe1A—C5A—C4A	119.7 (4)	C2B—Fe1B—C5B—C1B	−37.5 (3)
C8A—Fe1A—C5A—C4A	−42.6 (5)	C10B—Fe1B—C5B—C1B	116.1 (3)
C9A—Fe1A—C5A—C4A	−80.5 (3)	C9B—Fe1B—C5B—C1B	158.9 (3)
C10A—Fe1A—C5A—C4A	−124.1 (3)	C6B—Fe1B—C5B—C1B	74.2 (3)
C7A—Fe1A—C5A—C4A	155.7 (5)	C7B—Fe1B—C5B—C1B	39.5 (7)
C6A—Fe1A—C5A—C4A	−166.8 (2)	C8B—Fe1B—C5B—C1B	−166.0 (4)
C2A—Fe1A—C6A—C7A	−80.0 (3)	C3B—Fe1B—C6B—C10B	−166.8 (4)
C3A—Fe1A—C6A—C7A	−45.8 (5)	C4B—Fe1B—C6B—C10B	44.7 (7)
C1A—Fe1A—C6A—C7A	−122.9 (2)	C2B—Fe1B—C6B—C10B	159.3 (3)
C8A—Fe1A—C6A—C7A	37.4 (2)	C9B—Fe1B—C6B—C10B	−37.6 (3)
C9A—Fe1A—C6A—C7A	81.3 (3)	C7B—Fe1B—C6B—C10B	−119.1 (4)
C10A—Fe1A—C6A—C7A	118.9 (3)	C8B—Fe1B—C6B—C10B	−81.7 (3)
C5A—Fe1A—C6A—C7A	−163.5 (2)	C1B—Fe1B—C6B—C10B	117.0 (3)
C4A—Fe1A—C6A—C7A	163.9 (6)	C5B—Fe1B—C6B—C10B	76.1 (3)
C2A—Fe1A—C6A—C10A	161.1 (2)	C3B—Fe1B—C6B—C7B	−47.7 (6)
C3A—Fe1A—C6A—C10A	−164.6 (4)	C4B—Fe1B—C6B—C7B	163.9 (6)
C1A—Fe1A—C6A—C10A	118.3 (2)	C2B—Fe1B—C6B—C7B	−81.6 (3)
C8A—Fe1A—C6A—C10A	−81.5 (3)	C10B—Fe1B—C6B—C7B	119.1 (4)
C9A—Fe1A—C6A—C10A	−37.6 (2)	C9B—Fe1B—C6B—C7B	81.5 (3)
C5A—Fe1A—C6A—C10A	77.6 (3)	C8B—Fe1B—C6B—C7B	37.5 (3)
C7A—Fe1A—C6A—C10A	−118.9 (3)	C1B—Fe1B—C6B—C7B	−123.9 (3)
C4A—Fe1A—C6A—C10A	45.0 (7)	C5B—Fe1B—C6B—C7B	−164.8 (3)
C10A—C6A—C7A—C8A	0.7 (5)	C10B—C6B—C7B—C8B	−0.1 (5)
Fe1A—C6A—C7A—C8A	−58.7 (3)	Fe1B—C6B—C7B—C8B	−59.4 (3)
C10A—C6A—C7A—Fe1A	59.4 (3)	C10B—C6B—C7B—Fe1B	59.2 (3)
C2A—Fe1A—C7A—C6A	117.0 (2)	C3B—Fe1B—C7B—C8B	−81.8 (3)
C3A—Fe1A—C7A—C6A	159.0 (2)	C4B—Fe1B—C7B—C8B	−49.1 (5)
C1A—Fe1A—C7A—C6A	76.4 (3)	C2B—Fe1B—C7B—C8B	−124.6 (3)
C8A—Fe1A—C7A—C6A	−120.1 (3)	C10B—Fe1B—C7B—C8B	81.5 (3)
C9A—Fe1A—C7A—C6A	−82.2 (2)	C9B—Fe1B—C7B—C8B	37.7 (3)
C10A—Fe1A—C7A—C6A	−38.0 (2)	C6B—Fe1B—C7B—C8B	119.4 (4)
C5A—Fe1A—C7A—C6A	48.8 (6)	C1B—Fe1B—C7B—C8B	−165.3 (3)
C4A—Fe1A—C7A—C6A	−169.7 (4)	C5B—Fe1B—C7B—C8B	164.4 (6)
C2A—Fe1A—C7A—C8A	−122.9 (3)	C3B—Fe1B—C7B—C6B	158.8 (3)
C3A—Fe1A—C7A—C8A	−80.9 (3)	C4B—Fe1B—C7B—C6B	−168.5 (4)
C1A—Fe1A—C7A—C8A	−163.6 (2)	C2B—Fe1B—C7B—C6B	116.0 (3)
C9A—Fe1A—C7A—C8A	37.9 (2)	C10B—Fe1B—C7B—C6B	−37.9 (3)
C10A—Fe1A—C7A—C8A	82.1 (3)	C9B—Fe1B—C7B—C6B	−81.7 (3)
C5A—Fe1A—C7A—C8A	168.9 (5)	C8B—Fe1B—C7B—C6B	−119.4 (4)
C6A—Fe1A—C7A—C8A	120.1 (3)	C1B—Fe1B—C7B—C6B	75.3 (3)
C4A—Fe1A—C7A—C8A	−49.7 (5)	C5B—Fe1B—C7B—C6B	45.0 (7)
C6A—C7A—C8A—C9A	−0.7 (5)	C6B—C7B—C8B—C9B	0.2 (5)
Fe1A—C7A—C8A—C9A	−59.6 (3)	Fe1B—C7B—C8B—C9B	−59.0 (3)
C6A—C7A—C8A—Fe1A	58.9 (3)	C6B—C7B—C8B—Fe1B	59.2 (3)
C2A—Fe1A—C8A—C9A	−165.8 (2)	C3B—Fe1B—C8B—C7B	116.0 (3)
C3A—Fe1A—C8A—C9A	−125.0 (3)	C4B—Fe1B—C8B—C7B	158.5 (3)
C1A—Fe1A—C8A—C9A	164.8 (5)	C2B—Fe1B—C8B—C7B	74.1 (3)
C10A—Fe1A—C8A—C9A	37.9 (2)	C10B—Fe1B—C8B—C7B	−81.8 (3)
C5A—Fe1A—C8A—C9A	−52.6 (5)	C9B—Fe1B—C8B—C7B	−119.6 (4)
C7A—Fe1A—C8A—C9A	119.0 (4)	C6B—Fe1B—C8B—C7B	−37.8 (3)
C6A—Fe1A—C8A—C9A	81.8 (3)	C1B—Fe1B—C8B—C7B	42.0 (7)
C4A—Fe1A—C8A—C9A	−83.3 (3)	C5B—Fe1B—C8B—C7B	−168.4 (4)
C2A—Fe1A—C8A—C7A	75.2 (3)	C3B—Fe1B—C8B—C9B	−124.4 (3)
C3A—Fe1A—C8A—C7A	116.0 (3)	C4B—Fe1B—C8B—C9B	−81.9 (3)
C1A—Fe1A—C8A—C7A	45.9 (6)	C2B—Fe1B—C8B—C9B	−166.3 (3)
C9A—Fe1A—C8A—C7A	−119.0 (4)	C10B—Fe1B—C8B—C9B	37.7 (3)
C10A—Fe1A—C8A—C7A	−81.1 (3)	C6B—Fe1B—C8B—C9B	81.8 (3)
C5A—Fe1A—C8A—C7A	−171.6 (4)	C7B—Fe1B—C8B—C9B	119.6 (4)
C6A—Fe1A—C8A—C7A	−37.2 (2)	C1B—Fe1B—C8B—C9B	161.6 (5)
C4A—Fe1A—C8A—C7A	157.7 (2)	C5B—Fe1B—C8B—C9B	−48.9 (6)
C7A—C8A—C9A—C10A	0.4 (5)	C7B—C8B—C9B—C10B	−0.2 (5)
Fe1A—C8A—C9A—C10A	−59.2 (3)	Fe1B—C8B—C9B—C10B	−59.3 (3)
C7A—C8A—C9A—Fe1A	59.6 (3)	C7B—C8B—C9B—Fe1B	59.2 (3)
C2A—Fe1A—C9A—C8A	39.1 (6)	C3B—Fe1B—C9B—C10B	−165.9 (3)
C3A—Fe1A—C9A—C8A	73.3 (3)	C4B—Fe1B—C9B—C10B	−124.1 (3)
C1A—Fe1A—C9A—C8A	−167.5 (4)	C2B—Fe1B—C9B—C10B	159.6 (6)
C10A—Fe1A—C9A—C8A	−119.1 (4)	C6B—Fe1B—C9B—C10B	37.8 (3)
C5A—Fe1A—C9A—C8A	157.8 (3)	C7B—Fe1B—C9B—C10B	81.8 (3)
C7A—Fe1A—C9A—C8A	−37.9 (2)	C8B—Fe1B—C9B—C10B	119.1 (4)
C6A—Fe1A—C9A—C8A	−81.6 (3)	C1B—Fe1B—C9B—C10B	−47.1 (5)
C4A—Fe1A—C9A—C8A	115.2 (3)	C5B—Fe1B—C9B—C10B	−81.8 (3)
C2A—Fe1A—C9A—C10A	158.2 (5)	C3B—Fe1B—C9B—C8B	75.0 (3)
C3A—Fe1A—C9A—C10A	−167.6 (3)	C4B—Fe1B—C9B—C8B	116.8 (3)
C1A—Fe1A—C9A—C10A	−48.4 (5)	C2B—Fe1B—C9B—C8B	40.5 (7)
C8A—Fe1A—C9A—C10A	119.1 (4)	C10B—Fe1B—C9B—C8B	−119.1 (4)
C5A—Fe1A—C9A—C10A	−83.1 (3)	C6B—Fe1B—C9B—C8B	−81.3 (3)
C7A—Fe1A—C9A—C10A	81.2 (3)	C7B—Fe1B—C9B—C8B	−37.4 (3)
C6A—Fe1A—C9A—C10A	37.5 (2)	C1B—Fe1B—C9B—C8B	−166.2 (4)
C4A—Fe1A—C9A—C10A	−125.7 (3)	C5B—Fe1B—C9B—C8B	159.1 (3)
C7A—C6A—C10A—C9A	−0.5 (5)	C8B—C9B—C10B—C6B	0.1 (5)
Fe1A—C6A—C10A—C9A	59.0 (3)	Fe1B—C9B—C10B—C6B	−59.3 (3)
C7A—C6A—C10A—C11A	176.4 (4)	C8B—C9B—C10B—C11B	−178.7 (4)
Fe1A—C6A—C10A—C11A	−124.1 (4)	Fe1B—C9B—C10B—C11B	121.9 (4)
C7A—C6A—C10A—Fe1A	−59.5 (3)	C8B—C9B—C10B—Fe1B	59.4 (3)
C8A—C9A—C10A—C6A	0.1 (5)	C7B—C6B—C10B—C9B	0.0 (5)
Fe1A—C9A—C10A—C6A	−59.1 (3)	Fe1B—C6B—C10B—C9B	59.4 (3)
C8A—C9A—C10A—C11A	−176.8 (4)	C7B—C6B—C10B—C11B	178.8 (4)
Fe1A—C9A—C10A—C11A	124.0 (4)	Fe1B—C6B—C10B—C11B	−121.8 (4)
C8A—C9A—C10A—Fe1A	59.2 (3)	C7B—C6B—C10B—Fe1B	−59.3 (3)
C2A—Fe1A—C10A—C6A	−43.3 (5)	C3B—Fe1B—C10B—C9B	41.8 (7)
C3A—Fe1A—C10A—C6A	156.7 (6)	C4B—Fe1B—C10B—C9B	75.6 (3)
C1A—Fe1A—C10A—C6A	−80.4 (3)	C2B—Fe1B—C10B—C9B	−165.8 (4)
C8A—Fe1A—C10A—C6A	81.9 (3)	C6B—Fe1B—C10B—C9B	−119.3 (4)
C9A—Fe1A—C10A—C6A	119.6 (3)	C7B—Fe1B—C10B—C9B	−81.5 (3)
C5A—Fe1A—C10A—C6A	−123.4 (3)	C8B—Fe1B—C10B—C9B	−37.9 (3)
C7A—Fe1A—C10A—C6A	37.8 (2)	C1B—Fe1B—C10B—C9B	159.5 (3)
C4A—Fe1A—C10A—C6A	−165.8 (3)	C5B—Fe1B—C10B—C9B	116.9 (3)
C2A—Fe1A—C10A—C9A	−162.9 (4)	C3B—Fe1B—C10B—C6B	161.1 (6)
C3A—Fe1A—C10A—C9A	37.0 (7)	C4B—Fe1B—C10B—C6B	−165.0 (3)
C1A—Fe1A—C10A—C9A	159.9 (3)	C2B—Fe1B—C10B—C6B	−46.5 (5)
C8A—Fe1A—C10A—C9A	−37.8 (3)	C9B—Fe1B—C10B—C6B	119.3 (4)
C5A—Fe1A—C10A—C9A	117.0 (3)	C7B—Fe1B—C10B—C6B	37.8 (3)
C7A—Fe1A—C10A—C9A	−81.8 (3)	C8B—Fe1B—C10B—C6B	81.4 (3)
C6A—Fe1A—C10A—C9A	−119.6 (3)	C1B—Fe1B—C10B—C6B	−81.2 (3)
C4A—Fe1A—C10A—C9A	74.5 (3)	C5B—Fe1B—C10B—C6B	−123.8 (3)
C2A—Fe1A—C10A—C11A	76.5 (6)	C3B—Fe1B—C10B—C11B	−78.6 (8)
C3A—Fe1A—C10A—C11A	−83.5 (7)	C4B—Fe1B—C10B—C11B	−44.8 (5)
C1A—Fe1A—C10A—C11A	39.4 (4)	C2B—Fe1B—C10B—C11B	73.8 (6)
C8A—Fe1A—C10A—C11A	−158.3 (4)	C9B—Fe1B—C10B—C11B	−120.4 (5)
C9A—Fe1A—C10A—C11A	−120.5 (5)	C6B—Fe1B—C10B—C11B	120.3 (5)
C5A—Fe1A—C10A—C11A	−3.5 (4)	C7B—Fe1B—C10B—C11B	158.1 (5)
C7A—Fe1A—C10A—C11A	157.6 (4)	C8B—Fe1B—C10B—C11B	−158.3 (5)
C6A—Fe1A—C10A—C11A	119.8 (5)	C1B—Fe1B—C10B—C11B	39.1 (5)
C4A—Fe1A—C10A—C11A	−46.0 (4)	C5B—Fe1B—C10B—C11B	−3.5 (5)
N2A—N1A—C11A—C10A	−174.7 (4)	N2B—N1B—C11B—C10B	−176.5 (4)
C6A—C10A—C11A—N1A	0.7 (6)	C9B—C10B—C11B—N1B	178.3 (4)
C9A—C10A—C11A—N1A	177.0 (4)	C6B—C10B—C11B—N1B	−0.3 (7)
Fe1A—C10A—C11A—N1A	−91.0 (5)	Fe1B—C10B—C11B—N1B	−91.0 (5)
C13A—N3A—C12A—N2A	178.3 (4)	C13B—N3B—C12B—N2B	−177.3 (5)
C13A—N3A—C12A—S1A	−1.4 (6)	C13B—N3B—C12B—S1B	4.3 (7)
N1A—N2A—C12A—N3A	6.2 (6)	N1B—N2B—C12B—N3B	7.1 (6)
N1A—N2A—C12A—S1A	−174.1 (3)	N1B—N2B—C12B—S1B	−174.4 (3)
C12A—N3A—C13A—C14A	−175.2 (4)	C12B—N3B—C13B—C14B	84.8 (6)

### Hydrogen-bond geometry (Å, °)

**Table:**

*D*—H···*A*	*D*—H	H···*A*	*D*···*A*	*D*—H···*A*
N2A—H2NA···S1A^i^	0.82 (6)	2.59 (6)	3.387 (4)	164 (5)
N2B—H2NB···S1B^ii^	0.89 (9)	2.55 (9)	3.430 (5)	170 (5)
C4A—H4AA···S1B^iii^	0.98	2.79	3.715 (4)	157

Symmetry codes: (i) −*x*+1, −*y*+1, −*z*+1; (ii) −*x*, −*y*, −*z*; (iii) *x*, *y*, *z*+1.
